# Seroprevalence of West Nile, Rift Valley, and sandfly arboviruses in Hashimiah, Jordan.

**DOI:** 10.3201/eid0604.000405

**Published:** 2000

**Authors:** A Batieha, E K Saliba, R Graham, E Mohareb, Y Hijazi, P Wijeyaratne

**Affiliations:** Jordan University of Science and Technology, Irbid, Jordan. batieha@just.edu.jo

## Abstract

We conducted a serosurvey among patients of a health center in Hashimiah, a Jordanian town of 30,000 inhabitants located near a wastewater treatment plant and its effluent channel. Serum samples from 261 patients >/=5 years of age were assessed for immunoglobulin G (IgG) and IgM antibodies against West Nile, sandfly Sicilian, sandfly Naples, and Rift Valley viruses; the seroprevalence of IgG antibodies was 8%, 47%, 30%, and 0%, respectively. Female participants were more likely to have been infected than male. Persons living within 2 km of the treatment plant were more likely to have been infected with West Nile (p=0.016) and sandfly Sicilian (p=0.010) viruses. Raising domestic animals within the house was a risk factor for sandfly Sicilian (p=0.003) but not for sandfly Naples virus (p=0.148). All serum samples were negative for IgM antibodies against the tested viruses. Our study is the first documentation of West Nile and sandfly viruses in Jordan and calls attention to the possible health hazards of living close to wastewater treatment plants and their effluent channels.


### Seroprevalence of West Nile, Rift Valley, and Sandfly Arboviruses in Hashimiah, Jordan

**Anwar Batieha,* Elias K. Saliba,† Ross Graham,‡ Emad Mohareb,† Younis Hijazi,* and Pandu Wijeyaratne§**
*Jordan University of Science and Technology, Irbid, Jordan;
†Hashimiah University, Zarka, Jordan; ‡Virology Research Program, U.S. Naval Medical Research Unit No. 3, Cairo, Egypt;
§Infectious Disease Program of Nepal, USAID/EHP.

We conducted a serosurvey among patients of a health center in Hashimiah, a Jordanian town of 30,000 inhabitants located near a wastewater treatment plant and its effluent channel. Serum samples from 261 patients ≥5 years of age were assessed for immunoglobulin G (IgG) and IgM antibodies against West Nile, sandfly Sicilian, sandfly Naples, and Rift Valley viruses; the seroprevalence of IgG antibodies was 8%, 47%, 30%, and 0%, respectively. Female participants were more likely to have been infected than male. Persons living within 2 km of the treatment plant were more likely to have been infected with West Nile (p=0.016) and sandfly Sicilian (p=0.010) viruses. Raising domestic animals within the house was a risk factor for sandfly Sicilian (p=0.003) but not for sandfly Naples virus (p=0.148). All serum samples were negative for IgM antibodies against the tested viruses. Our study is the first documentation of West Nile and sandfly viruses in Jordan and calls attention to the possible health hazards of living close to wastewater treatment plants and their effluent channels.

Arboviruses are transmitted by arthropods. Humans become infected through the bites of blood-sucking insects such as mosquitoes, ticks, and certain flies. The geographic distribution of the viruses varies with the presence and density of the appropriate vector. In Jordan, there have been no previous studies of arboviral infections and no clinical reports of the existence of such infections. We report the findings of a serosurvey of West Nile, sandfly Sicilian, sandfly Naples, and Rift Valley viruses. The study area has an abundance of *Culex pipiens* mosquitoes, which breed in the effluent channel of a nearby wastewater treatment plant (1). The presence of the above viruses in neighboring countries and the abundance of their vectors in the study area were the reasons the viruses were selected for this study.

Address for corrrespondence: Anwar Batieha, Jordan University of Science and Technology, P.O. Box 3030, Irbid, Jordan; fax 962-2-7095123; e-mail: batieha@just.edu.jo.

### Methods

### Study site

Hashimiah (32°7'N and 36°6'E) is a town of approximately 30,000 inhabitants in close proximity to a wastewater treatment plant and its effluent channel. The plant uses stabilization ponds, which receive more than double the amount of wastewater they were designed to treat. This overload results in insufficient treatment and poor-quality effluent. The effluent channel has many areas with excessive vegetation and stagnant water. Local residents have reported high mosquito and sandfly density and bad odor in the area. A baseline health assessment was undertaken as part of a comprehensive effort to solve the problem.

### Study population

A total of 501 persons ≥5 years of age who attended the local health center from June 20 to July 30, 1998, were invited to participate. Of those, 261 (52%) agreed to undergo all the study procedures, including giving a blood sample.

Children <5 years of age were excluded because of the technical difficulties of drawing blood samples and obtaining their guardians' consents.

### Data collection

Each participant was interviewed by use of a structured questionnaire, including questions about sociodemographic and other variables related to exposure to mosquitoes and domestic animals, and had a general physical examination focusing on pest-related problems. A 10-mL venous blood sample, obtained from each participant, was separated within 3 hours of collection and stored in an ice bag at -20°C until transported to Cairo, Egypt, for final laboratory analysis.

Samples were tested in U.S. Naval Medical Research Unit No 3 (NAMRU-3) laboratories. An enzyme-linked immunosorbent assay (ELISA) was performed for immunoglobulin G (IgG) and IgM antibodies against West Nile, sandfly Sicilian, sandfly Naples, and Rift Valley viruses. The West Nile strain was EG101, which was passed in mice (14 times) and in Vero cells (3 times). The two sandfly viruses were Sabin strain. Sicilian virus was passed in mice (37 times) and in Vero cells (4 times); Naples virus was passed in mice (49 times) and in Vero cells (4 times). The Rift Valley strain was ZH-501, which was passed in mice (6 times), in E-6 cells (once), and in Vero cells (3 times). A standard direct IgG ELISA was used for virus antigens. An IgM-capture assay employing anti-human IgM (Kirkegaard & Perry Laboratories, Inc., Gaithersburg, MD, USA) was used for the IgM assays. The viruses were grown in tissue cultures. At approximately 50% cytopathic effect, the viral proteins were extracted with Triton X (1%). These virus lysates were used for both IgG and IgM ELISAs. ELISAs for IgG used 96-well plates coated with antigens (viral infected and uninfected Vero cells) extracted with 1% Triton X (Sigma T-9284). Serum samples were added to the plates, and bound antibodies were detected by using goat anti-human IgG conjugated to horseradish peroxidase and detected with ABTS (2,2'-Azinopis [3-ethylbenzothiazoline-6-sulphonic acid], diammonium salt) substrate. For IgM, goat anti-human IgM antibodies were absorbed to ELISA plates and used to capture the patient's serum IgM. Anti-virus-specific IgM was detected by using the antigens and hyperimmune mouse serum. The antigen-antibody complexes were detected using goat anti-mouse IgG-horseradish peroxidase and ABTS substrate. All conjugates, capture IgM antibodies, and ABTS were from Kirkegaard & Perry.

### Ethical Considerations

The study was undertaken in response to public concerns regarding potential health hazards of the wastewater treatment plant and its effluent channel on neighboring residents. The study protocol was approved by the Jordanian Ministry of Health. Verbal consent was obtained from all participants or their legal guardians. All identifying information was kept confidential.

### Data Management and Statistical Analysis

Data entry and analysis used Epi-Info, version 6 software (2). Seropositivity was determined by a number of variables. Observed differences were assessed for statistical significance by chi-square, corrected for continuity.

### Results

### West Nile Virus

Approximately 8% of the study participants had evidence of past infection with West Nile virus (Table 1). Although information on travel

Table 1. Seropositivity of immunoglobulin G antibodies against West Nile virus, Jordan, 1998

| Variable | Total | Seropositivity N (%) | p |
|---|---|---|---|
| Total | 261 | 21 (8.0) | |
| Sex | | | 0.202 |
| Male | 75 | 3 (4) | |
| Female | 186 | 18 (9.7) | |
| Age | | | 0.920 |
| 5-9 years | 14 | 1 ( 7.1) | |
| 10-29 years | 158 | 12 ( 7.6) | |
| ≥30 years | 89 | 8 ( 9.0) | |
| Monthly family income | | | 0.284 |
| <100 JD[a] | 76 | 9 (11.8) | |
| 100-249 JD | 156 | 11 ( 7.1) | |
| ≥250 JD | 29 | 1 ( 3.5) | |
| Presence of domestic animals | | | 0.578 |
| In house | 86 | 9 (10.5) | |
| Near house | 24 | 2 ( 8.3) | |
| None | 151 | 10 ( 6.6) | |
| Distance from plant | | | 0.016 |
| Residence within 2 km | 115 | 15 (13.0) | |
| More than 2 km | 146 | 5 ( 4.1) | |
| Presence of mosquito bites on exam | | | 0.660 |
| Yes | 156 | 14 ( 9.0) | |
| No | 105 | 7 ( 6.7) | |

[a]JD = Jordanian dinars.

was not collected, mobility of the study population is limited and thus unlikely to be the cause of infection with the virus. Cross-reactivity to other related flaviviruses is unlikely since no such viruses have been documented in Jordan. The infection rate among female participants (9.7%) was more than double that among male (4.0%), but it was not statistically significant (p=0.202). Although older age (≥30 years), lower family income (<100 Jordanian dinars), presence of domestic animals within the house, and presence of mosquito bites on examination seemed to be related to a higher prevalence of past infection with West Nile virus, none of these variables had significant effect. The only significant factor for past infection was distance between residence and treatment plant and its effluent channel. Study participants living within 2 km were approximately 4 times more likely to have been infected than participants living further away (p=0.016). No participants had evidence of acute infection with West Nile virus.

### Sandfly Sicilian Virus

More than 47% of the study population had evidence of past infection with sandfly Sicilian virus (IgG seropositivity, Table 2). Female sex, presence of domestic animals within the house, and close residence to the treatment plant and its effluent channel were significantly associated with a higher prevalence of past infection with sandfly Sicilian virus. There was no evidence of acute infection (IgM positivity) with sandfly Sicilian virus.

### Sandfly Naples Virus

More than 29% of the participants had IgG antibodies against sandfly Naples virus (Table 3). The only factor significantly related to past infection was age: participants ≥30 years of age were more likely to have been infected than those in the younger age groups (p=0.007); all were IgM seronegative, which indicates absence of acute infection with this virus.

### Rift Valley Virus

All participants were seronegative for IgG and IgM antibodies against Rift Valley virus, which indicates that the study population had never been exposed to the virus.

### Discussion

Our study is the first documentation that West Nile, sandfly Sicilian, and sandfly Naples

Table 2. Seropositivity of immunoglobulin G antibodies against sandfly Sicilian virus, Jordan, 1998

| Variable | Total | Seropositivity N (%) | p |
|---|---|---|---|
| Total | 261 | 123 (47.1) | |
| Sex | | | 0.003 |
| Male | 75 | 24 (32.0) | |
| Female | 186 | 99 (53.2) | |
| Age | | | 0.284 |
| <10 years | 14 | 6 (42.9) | |
| 10-29 | 158 | 69 (43.7) | |
| ≥30 | 89 | 48 (53.9) | |
| Monthly family income | | | 0.277 |
| <100 JD[a] | 76 | 41 (53.9) | |
| 100-249 JD | 156 | 71 (45.5) | |
| ≥250 JD | 29 | 11 (37.9) | |
| Presence of domestic animals | | | 0.003 |
| In house | 86 | 53 (61.6) | |
| Near house | 24 | 8 (33.3) | |
| None | 151 | 62 (41.1) | |
| Distance from plant | | | 0.010 |
| Residence within 2 km | 115 | 65 (56.5) | |
| More than 2 km | 146 | 58 (39.7) | |
| Presence of mosquito bites on exam | | | 0.804 |
| Yes | 156 | 75 (48.1) | |
| No | 105 | 48 (45.7) | |

[a]JD = Jordanian dinars.

Table 3. Seropositivity of immunoglobulin G antibodies against sandfly Naples virus, Jordan, 1998

| Variable | Total | Seropositivity N (%) | p |
|---|---|---|---|
| Total | 261 | 77 (29.5) | |
| Sex | | | 0.165 |
| Male | 75 | 17 (22.7) | |
| Female | 186 | 60 (32.3) | |
| Age | | | 0.007 |
| <10 years | 14 | 1 ( 7.1) | |
| 10-29 | 158 | 40 (25.3) | |
| ≥30 | 89 | 36 (40.4) | |
| Monthly family income | | | 0.68 |
| <100 JD[a] | 76 | 24 (31.6) | |
| 199-249 JD | 156 | 43 (27.6) | |
| ≥250 JD | 29 | 10 (34.5) | |
| Presence of domestic animals | | | 0.148 |
| In house | 86 | 32 (37.2) | |
| Near house | 24 | 7 (29.2) | |
| None | 151 | 38 (25.2) | |
| Distance from plant | | | 0.667 |
| Residence within 2 km | 115 | 36 (31.3) | |
| More than 2km | 146 | 41 (28.1) | |
| Presence of mosquito bites on exam | | | 0.485 |
| Yes | 156 | 43 (27.6) | |
| No | 105 | 34 (32.4) | |

[a]JD = Jordanian dinars.

viral infections are present in Jordan. The prevalence of sandfly viral infection was much higher than that of West Nile, but no acute sandfly infections were detected, possibly because 1) IgM positivity is short-lived and therefore the chance to be detected in a survey is much smaller and 2) children ≤5 years of age (who are more likely to be susceptible for acute infection) were excluded from the study.

Humans become infected with West Nile virus by the bite of an infected *Culex* mosquito. *C. pipiens* are abundant in the study area (1). Birds are the reservoirs of infection (3). The presence of the disease in Jordan is not unexpected as its known geographic distribution includes the Middle East, Africa, southern Europe, and Asia (4). In 1989, the seroprevalence of IgG antibodies to this virus among schoolchildren ages 8 to 14 years was 3% in an area in the Nile River Delta (5). In another report from Egypt, the seroprevalence of West Nile virus antibodies was 20% (6). In 1996, an epidemic of 393 cases of West Nile meningoencephalitis occurred in Romania (7). Recently, and for the first time in the United States, an outbreak of West Nile-like encephalitis occurred in New York (8, 9).

Infection with sandfly viruses is transmitted by the bites of infected *Phlebotomus pappatasi* sandflies. The principal reservoirs are humans and sandflies (10), though rodents are suspected to harbor them (3). Aseptic meningitis caused by Sicilian virus has been reported (11). Sandfly fever is present in the circum-Mediterranean area, extending to the east through the Balkans into China, the Middle East, and Southwest Asia (4). Travelers to disease-endemic areas and deployed troops are at high risk of contracting the disease (12). Among 298 Swedish United Nations soldiers who served in Cyprus, seroconversion, in a 6-month period, occurred in 7 (for Sicilian virus), 3 (Naples), and 1 (Toscana) (13). Cyprus seems to have a high prevalence of sandfly viruses, with a reported seroprevalence rate of 57% for Sicilian, 32% for Naples, and 20% for Toscana virus (14). Sandfly Sicilian and Naples virus infections have been documented in Egypt (5,6). Although these viruses have not been reported in Jordan, their vector (*P. pappatasi*) is ubiquitous (15-18), including in the Hashimiah area (Saliba EK, unpublished data). Sandflies breed mainly in dirt and garbage, but not in wastewater. The high prevalence of both sandfly fever viruses in Hashimiah may be attributed to the fact that immunity is serotype specific, i.e., infection with one serotype provides no protection for the other (12).

Possible explanations for the higher infection rates among women are their likelihood of spending most of their time at home and their caring for domestic animals in places not protected from mosquitoes and sandflies. These factors may also explain the higher prevalence of past infection among lower income people, who live close to the plant and its effluent channel, are more likely to raise domestic animals (mostly sheep and cows), and often keep the animals inside the homes. These circumstances create environmental conditions suitable for sandfly breeding.

Unlike sandfly Sicilian, the associations of sandfly Naples with gender, presence of domestic animals, and distance from the plant were not significant. The smaller number of seropositive samples for sandfly Naples may explain these inconsistencies. On the other hand, the higher endemicity of the Sicilian virus leads to exposure and subsequent immunity at an earlier age than for the Naples virus. Because young children (≤5 years) were excluded from the study, a weaker association between the Sicilian virus and age is not unexpected.

With the unprecedented increased population mobility in the form of tourism, business, and troop deployment, political borders are no longer barriers against the spread of infections. The West Nile-like encephalitis outbreak in New York, which is likely to have been transmitted from the Middle East (19), provides viable support for this notion. Therefore, our findings may be of interest outside, as well as within, Jordan. At the local level, the data should alert physicians to consider these viral infections in the differential diagnosis of conditions such as encephalitis, aseptic meningitis, and unexplained febrile illnesses. The data also highlight the need for preventive measures, such as educating people about self-protection and instituting public health programs directed against mosquitoes and sandflies. The study also calls attention to the possible health hazards of wastewater plants and, in particular, their effluent channels, on neighboring communities. Wastewater effluent channels, if not well maintained, provide potential breeding sites for *C. pipiens*. The absence of Rift Valley virus infection among the studied population does not mean it is absent in other areas in Jordan. Further studies in different geographic areas are recommended.

### Acknowledgments

We thank Fred Rosensweig for his encouragement; Mohammad Al-Haziemeh and the staff of Al-Hashimiah Health Center for their cooperation and help; and Al-Hashimiah residents for their participation.

The work reported in this article was funded by the U.S. Agency for International Development through the Environmental Health Project, a USAID contractor sponsored by the Office of Health and Nutrition, Bureau for Global Programs, Field Support and Research (Contract No. HRN-C-00-93-0036-11).

Dr. Batieha is a physician and epidemiologist currently serving as associate professor of epidemiology at Jordan University of Science and Technology, Irbid, Jordan. Research interests are wide, including cancer and micronutrients, cardiovascular risk factors, tuberculosis, and infectious diseases.

### References

1. Wijeyaratne P. Initial investigation of insects and other pests in communities around the As-Samra Waste Water Treatment Plant. Environmental Health Project. Washington: U.S. Agency for International Development. Report for the file No. 140; 1997.
2. Dean AG, Dean JA, Coulombier D, Brendel KA, Smith DC, Burton AH, et al. Epi-Info, Version 6: a word-processing, database, and statistics program for public health on IBM-compatible microcomputers. Atlanta (Ga):Centers for Disease Control and Prevention;1995.
3. Monath TP, ed. Arboviruses: epidemiology and ecology. Elkins Park (PA): Franklin Book Company, 1989.
4. Peters CJ. Infections caused by arthropod- and rodent-borne viruses. In: Fauci AS, Braunwald E, Isselbacher KJ, Wilson JD, Martin JB, Kasper DL, eds. Harrison's Principles of Internal Medicine. 14th ed. Vol. 1. New York: McGraw-Hill, 1998;1138.
5. Corwin A, Habib M, Olson J, Scott D, Ksiazek T, Watts DM. The prevalence of arboviral, rickettsial, and Hantaan-like viral antibody among school children in the Nile River Delta of Egypt. Trans R Soc Trop Med Hyg 1992;86:677-9.
6. Corwin A, Habib M, Watts D, Darwish M, Olson J, Botros B, et al. Community-based prevalence profile of arboviral, rickettsial, and Hantaan-like viral antibody in the Nile River Delta of Egypt. Am J Trop Med Hyg 1993;48:776-83.
7. Han LL, Popovici F, Alexander Jr JP, Laurentia V, Tengelsen LA, Cernescu C, et al. Risk factors for West Nile Fever virus infection and meningoencephalitis, Romania, 1996. J Infect Dis 1999;179:230-3.
8. Centers for Disease Control and Prevention. Outbreak of West Nile-like viral encephalitis—New York, 1999. MMWR Morb Mortal Wkly Rep 1999;48:845-9.
9. Centers for Disease Control and Prevention. Update: West Nile-like viral encephalitis—New York, 1999. MMWR Morb Mortal Wkly Rep 1999; 48:890-2.
10. Tesh RB, Modi GB. Studies on the biology of phleboviruses in sandflies (Diptera: Psychodidae). 1. experimental infection of the vector. Am J Trop Med Hyg 1981, 33:1007-16.
11. Becker M, Zielen S, Schwarz TF, Linde R, Hofmann D. Pappataci fever (abstract). Klin Padiatr 1997;209:377-9.
12. Tesh RB. The epidemiology of *Phlebotomus* (Sandfly) fever. Isr J Med Sci 1989;25:214-7.
13. Eitrem R, Vene S, Niklasson B. Incidence of sandfly fever among Swedish United Nations soldiers on Cyprus during 1985. Am J Trop Med Hyg 1990;43:207-11.
14. Eitrem R, Stylianou M, Niklasson B. High prevalence rates of antibody to three sandfly fever viruses (Sicilian, Naples, and Toscana) among Cypriots. Epidemiol Infect 1991;107:685-91.
15. Oumeish OY, Saliba EK, Allawi TF. Cutaneous leishmaniasis, an endemic disease in Jordan. Jordan Medical Journal 1982;15:55-61.
16. Janini R, Saliba E, Kamhawi S. Species composition of sandflies and population dynamics of *Phlebotomus pappatasi* (Diptera: Psychodidae) in an endemic focus of cutaneous leishmaniasis: the southern Jordan Valley. J Med Entomol 1995;32:822-6.
17. Kamhawi S, Abdel-Hafez SK, Molyneux DH. A comprehensive account of species composition, distribution and ecology of phlebotomine sandflies in Jordan. Parasite 1995;2:163-72.
18. Saliba EK, Saleh N, Oumeish OY, Khouri S, Bisharat Z, Al-Ouran R. The endemicity of leishmania tropica (zymodeme MON-137) in the Eira-Yarka area of Salt District, Jordan. Ann Trop Med Parasitol 1997;5:453-9.
19. Lanciotti RS, Roehrig JT, Deubel V, Smith J, Park Crise B, Volpe KE, et al. Origin of the West Nile virus responsible for an outbreak of encephalitis in the northeastern United States. Science 1999;286:2333-7.
